# Chicago Pathological Society

**Published:** 1891-03

**Authors:** 


### CHICAGO PATHOLOGICAL SOCIETY.

Regular monthly meeting, First Vice-President, Dr. E. H. Root, in the chair.

Dr. Joseph M. Patton read a paper on "Cardiac Arhythmia." By Cardiac Arhythmia, is meant some departure from the natural physiological rythm of the heart's action. Irregular heart's action, he said, is not a constant attendant of organic disease of the valves or the heart muscles; on the contrary, eliminating all nervous and reflex causes, it is seldom present in such conditions, except during exertion, or when a hyposystolic or asystolic condition has been developed, and is then evidence of mechanical inability of the heart muscle to perform its rhythmical action properly. The severity of the arythmia is not to be depended on as an indication of the non-integrity of the heart muscle. For instance, in a recent case of dilatation of the heart cavities with extensive pericordial adhesions, very severe arhythmia had been an enduring symptom a few hours before death, and as unconsciousness developed the heart became more regular even than it had been at any time for months previous.

There are many forms of arhythmia arising as a result of disturbances in other organs. Thus we often find it present in gastric catarrh, hyper-acidity of the stomach, inflammatory conditions of the duodenum and bile tracts, congestions of the liver, intestinal worms, ovarian diseases, early in pregnancy, etc. These forms of arhythmia probably arise from disturbance of the sympathetic nervous system, and the only reason we can give for their appearance in only a portion of cases where abdominal irritation exists, is that of unusual irritability of the cardiac plexuses and ganglia.

Concerning the pathology of the more purely nervous forms of arhythmia, these have been studied by Lancereaux, Iwanowski, Putjatkin, Uskoff and others, with reference to the nerve fibres and ganglia of the heart; they have found nuclear and connective tissue proliferations; degeneration of nerve cells, and fatty and pigmentary change in the ganglia. S. Mayer has shown that there are in the nerve fibres changes of degeneration and regeneration, which obtain in normal conditions, and this is probably true of the ganglia.

Among the nervous forms of arhythmia is that induced by the excessive use of tobacco. Fraentzel thinks that changes in the musculo-motor and inhibitory heart centers are probably the cause, and that it does not depend on the amount of nicotine taken into the system. On the latter point Dr. Patton cannot agree. While the amount of poison which will affect different individuals may vary greatly, the effect in any one individual who is the subject of a tobacco heart will be directly proportionate to the strength and amount of tobacco consumed, a point which the author quoted virtually admits. It is probable that the effect here is through disturbance of the accelerator centers, rather than paralysis of the inhibitory apparatus. This form of arhythmia is generally more or less intermittent, especially in the milder forms; a temporary indigestion is apt to induce or aggravate an arhythmia of this nature. There are no changes to be detected on physical examination, and if the use of tobacco is dispensed with, the heart generally will gradually return to a normal action, though sometimes a nervous arhythmia is induced which remains for years or during life.

Dr. Homer M. Thomas, in opening the discussion, said: Regarding the use of tobacco, Dr. Patton's statements so thoroughly coincide with my own experience that I was favorably impressed with them. My experience in the treatment of such affections, particularly in a large number of cases of irritable heart from the excessive use of tobacco, is that just in proportion as we lessen the amount of tobacco that they use, we diminish the cardiac arhythmia and restore the heart to a more normal action. The sounds will be more normal, the heart-beat stronger, and the general functional disturbance lessened.

In regard to arhythmia being dependent upon mitral obstruction, I have seen several cases that appeared to bear out that observation. There is just a slight murmur preceding the first sound of the heart. If the mitral disease develops into the regurgitant form, I have not noticed, nor do I recall a case in which you get arhythmia. There is a familiar form of cardiac arhythmia dependent upon gastric disorders, and in proportion as we are able to remove the exciting cause of this condition by directing our attention to the proper selection of foods, etc., we remove the irregular heart action.

I think the term "American heart" is a very apt expression for in no other nationality that I am familiar with do we get that form of irritable heart—railroad heart—that we do with the Americans. They have a most characteristic heart action, and it seems to coincide with their general nervous temperament.

Dr. J. E. Colburn: I would like to ask Dr. Patton whether or not he has examined a large number of cases to ascertain the average heart rate. For instance, take the American, German, French or English heart; of course we have the old rate of about 60 beats to the minute, but having had considerable experience in examining men for life insurance companies, I find that the average is over 75, and whether this high rate is due to the excitement attending the examination, I have not been able to satisfy myself. During a period of some eight or ten years, I have examined the heart in hundreds of cases, and I have been impressed with its high rate of action, which I formerly supposed to be normal.

Then, again, the value or possibility of making a diagnosis of cases of irregular heart action and its relation to life insurance. It seems to me that this is quite an important matter, and one that has and is receiving considerable attention from English physicians. We find some gentlemen saying that unless well-defined obstruction can be found, or there is evidence of an organic disease, that an irregular heart is a safe heart, and they will recommend men for insurance, while in this country they would be excluded. If an irregular heart is found to be due to the excessive use of tobacco, the applicant for life insurance is compelled to pledge a diminution or cessation of its use before the company will accept him.

Dr. E. H. Root: A question that has puzzled me not a little in cases of midwifery is, that instead of a rapid pulse, we get a slow one, it being sometimes as slow as 40 and 50. These have proved the most tedious cases of hemorrhage in my practice. I had to watch them very closely. Whether this condition is due to prostration from the life courses incident to delivery, I am not able to say. The hemorrhage is more profuse if the patient is not kept quiet. In a number of cases in which I have been called at the time of delivery, it was fully twenty-four hours before the heart was restored to a normal condition. Careful examination revealed no cardiac lesion. In one of my cases there was no internal hemorrhage, and very little blood was expelled externally. The next day a large clot from the uterus was expelled with a sharp contractile pain. I think that slowness of the pulse begins before delivrey very often, and continues through the second stage of labor, and hemorrhage is sure to occur in the third stage.

Dr. J. M. Patton: The peculiarity referred to by Dr. Root does not always correspond to the amount of blood lost by the patient during labor. A slow pulse is not due to the fact that the patient loses blood, but is probably due to the effect on the innervation of the heart which is produced by the shock incident to parturition. As Dr. Root has said, these are dangerous cases, and should be watched carefully.

In reply to Dr. Thomas, I would say that in mitral stenosis arhythmia is present only where there is simply a narrowing of the valve opening, and, as he has aptly said, that disappears when regurgitation is obtained. The arhythmia is a mechanical one, as far as it is possible for us to have a mechanical arhythmia of the heart, which is due to a mechanical change in the heart tissue. We deny the liability to have arhythmia from physical changes in the structure of the heart, except in marked conditions of a systole or the later stages of heart disease.

In answer to the question propounded by Dr. Colburn, I

would say that I have made no observations as regards the pulse rate in the ordinary healthy individual. There have been a large number of observations made by different men. Dr. Morton Prince, a year or so ago, made a tabulated statement of life insurance examinations as regards the relative frequency of the heart and endocardial murmurs especially in applicants for life insurance, and gave the pulse rates of most of the patients. I cannot recall what the average pulse rate was, but it was rather high. My experience with life insurance examinations is that the average male pulse will be from 75 to 80. We have to make allowance for the condition of the patient. There is not one man in fifty, who goes before a life insurance examiner, but what his pulse is five or six beats faster than at any other time. As regards the effect of the pulse rate and cardiac murmurs, I think our insurance companies in this country are wrong. If we state to a company that a man has something the matter with his heart, no matter what it is, the majority of them will reject him. The average man with irregularity of the heart, provided it is not due to the later stages of heart disease, is just as good a risk as a man with a regular heart. He is just as good a risk as a man who is liable to get nervous at times and leave his business to get fresh air simply because he is nervous and fidgety. It is simply a nervous manifestation. Why it is directed towards the heart we cannot always tell. Personally, I believe a great share of the men who are the subjects of even organic disease, are just as good risks in regard to time as the majority of men who are taken in without any organic disease. I know of men that have been under observation for ten years, who were rejected on account of a slight valvular lesion, and who are in just as good health to-day as they were then.

Some of the English life insurance companies have done away with medical examinations altogether, and their results are just as good as with them, according to their statements. It does not make much difference how good a man's judgment may be in regard to cardiac lesions. You may examine a man with some slight cardiac trouble who is a good risk as to time, yet the company will not accept him. I rejected a man on account of a slight mitral regurgitation, although I regarded him as a good risk. I wrote to the chief medical examiner and explained his business habits and his general physical condition, and the reason why I thought he was a good risk, and he wrote me he would not take him. Six months thereafter another medical examiner connected with the company declared that the man had no cardiac trouble, and he was accepted.

I do not wish to be understood as deprecating the necessity of medical examinations. The greater danger a cardiopathic person may sustain through the occurrence of acute diseases must be considered, even though his life expectancy, as far as the condition of the heart is concerned, may be as great as the man with a healthy organ. I think, however, that the prohibition placed upon irregular hearts by insurance companies, without distinction as to cause, unwarranted.

There are a great many things which interfere with the result of medical examinations, and until life insurance companies make a general ıevision of the laws governing such examinations, and their effect upon the life expectation of the applicant, I really think they can get along as well without as with medical aid.

A Member: I would like to ask Dr. Patton what the usual pulse rate is in case of hysteria?

Dr. Patton: In regard to hysteria, my experience is that the cardiac symptoms of hysterical manifestations are about as eccentric and unreliable as the others we encounter. They do not require much attention. Some patients we find with a very rapid heart or pulse beat, in others it is very slow. For instance, I saw a patient two weeks ago who was having violent hysterical paroxysms, and her pulse was as regular as mine A year ago, one night I saw a young woman who had a hysterical attack simulating angina pectoris, whose heart was very rapid at times, then very slow, so that it more closely resembled angina pectoris than anything you could imagine. If it had been in an older person, it would have been difficult to differentiate it from angina pectoris. In this instance the trouble was due to reflex ovarian irritation. As a matter of record, the heart symptoms are just as eccentric as the rest we meet with in hysterical manifestations.

